# Recombinant DNA production of spider silk proteins

**DOI:** 10.1111/1751-7915.12081

**Published:** 2013-10-11

**Authors:** Olena Tokareva, Valquíria A Michalczechen-Lacerda, Elíbio L Rech, David L Kaplan

**Affiliations:** 1 Department of Biomedical Engineering, Tufts UniversityMedford, MA, 02155, USA; 2 Department of Cell Biology, Campus Universitario Darcy Ribeiro, Institute of Biology, University of BrasiliaBrasilia, DF, 70910-900, Brazil; 3 Embrapa Genetics Resources and Biotechnology, Biotechnology UnitParque Estação Biológica PqEB W5 Norte, Brasilia, 70770-900, DF, Brazil

## Abstract

Spider dragline silk is considered to be the toughest biopolymer on Earth due to an extraordinary combination of strength and elasticity. Moreover, silks are biocompatible and biodegradable protein-based materials. Recent advances in genetic engineering make it possible to produce recombinant silks in heterologous hosts, opening up opportunities for large-scale production of recombinant silks for various biomedical and material science applications. We review the current strategies to produce recombinant spider silks.

## Introduction

**Figure 1:**
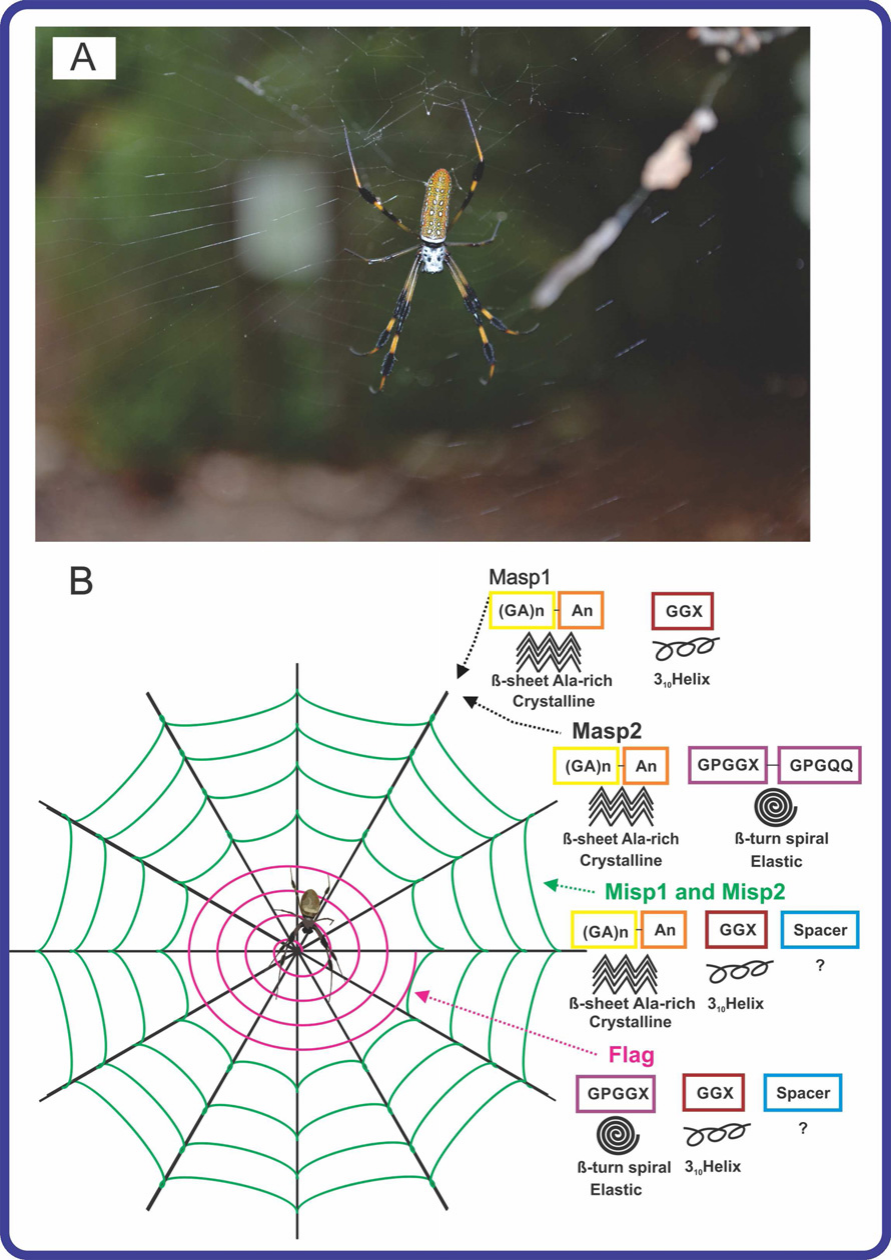
A. An adult female orb weaver spider *Nephila clavipes* and her web. B. Schematic overview of *N. clavipes* web composed of three different spider silk proteins and their structures. The coloured boxes indicate the structural motifs in silk proteins. An empty box marked ‘?’ indicates that the secondary structure of the ‘spacer’ region is unknown. Note: MaSp1 or MaSp2: major ampullate spidroin 1 or 2; MiSp1 and 2: minor ampullate spidroin1 and 2; Flag: flagelliform protein. The photo was taken by Olena and Artem Tokarev in the Florida Keys.

Spider silks have been a focus of research for almost two decades due to their outstanding mechanical and biophysical properties. Spider silks are remarkable natural polymers that consist of three domains: a repetitive middle core domain that dominates the protein chain, and non-repetitive N-terminal and C-terminal domains. The large core domain is organized in a block copolymer-like arrangement, in which two basic sequences, crystalline [poly(A) or poly(GA)] and less crystalline (GGX or GPGXX) polypeptides alternate. At least seven different types of silk proteins are known for one orb-weaver species of spider (Lewis, 2006a). Silks differ in primary sequence, physical properties and functions (Hu *et al*., 2006). For example, dragline silks used to build frames, radii and lifelines are known for outstanding mechanical properties including strength, toughness and elasticity (Gosline *et al*., 1984). On an equal weight basis, spider silk has a higher toughness than steel and Kevlar (Vepari and Kaplan, 2007; Heim *et al*., 2009). Flageliform silk found in capture spirals has extensibility of up to 500%. Minor ampullate silk, which is found in auxiliary spirals of the orb-web and in prey wrapping, possesses high toughness and strength almost similar to major ampullate silks, but does not supercontract in water. Figure 1 depicts the location and structural elements of MaSp, MiSp and Flag silks.

Finally, there are other silk types such as aciniform, pyriform, aggregate and tubuliform (egg case) with unusual primary structure, composition and properties. Diverse and unique biomechanical properties together with biocompatibility and a slow rate of degradation make spider silks excellent candidates as biomaterials for tissue engineering, guided tissue repair and drug delivery, for cosmetic products (e.g. nail and hair strengthener, skin care products), and industrial materials (e.g. nanowires, nanofibres, surface coatings).

Recent advances in genetic engineering have provided a route to produce various types of recombinant spider silks (Prince *et al*., 1995; Fahnestock and Bedzyk, 1997; Rabotyagova *et al*., 2009; Xu *et al*., 2007). However, production of spider silk proteins at a larger scale remains challenging. Moreover, recombinant silk threads do not recapitulate the full potential of native fibres in terms of mechanical properties. Different heterologous host systems have been investigated to develop suitable production systems. In this review, we discuss recent advances in the production of recombinant spider silks in heterologous host systems with the main focus on microbial production. In particular, we focus on dragline silks. Current cloning strategies, expression systems and purification strategies will be discussed to help researchers to engineer customized synthetic spider silk-like proteins for various needs, including biomaterials and material science applications.

### Structure of silk proteins

Spider silks are fascinating polymers, as is the spinning process that members of *Araneidae* family use to make these exceptional materials. Spiders use complex spinning to rapidly transform water soluble, high molecular weight, silk proteins into solid fibres at ambient temperature and pressure, giving rise to an environmentally safe, biodegradable and high performance material (Asakura *et al*., 2007; Lewicka *et al*., 2012; Teulé *et al*., 2012a). The details on anatomy and physiology of the spider spinning apparatus (*N. clavipes*) can be found elsewhere (Knight and Vollrath, 2001; 2002; Eisoldt *et al*., 2011; Rising *et al*., 2011).

In order to understand the challenges and needs associated with biotechnological production of recombinant spider silks, primary protein motifs, composition and secondary structural elements must be discussed. As mentioned earlier, one spider is capable of producing up to seven different types of silks with varying mechanical properties. In spite of different mechanical and physiological properties, the majority of spider silks share a common primary structural pattern comprised of a large central core of repetitive protein domains flanked by non-repetitive N- and C-terminal domains. The most investigated silk is dragline silk, which shows a remarkable combination of strength and elasticity. The golden orb-weaver spider, *N. clavipes*, produces dragline silk in the major ampullate gland (Knight and Vollrath, 2001). Dragline silk is the protein complex composed of major ampullate dragline silk protein 1 (MaSp1) and major ampullate dragline silk protein 2 (MaSp2). Both silks are approximately 3500 amino acid long. MaSp1 can be found in the fibre core and the periphery, whereas MaSp2 forms clusters in certain core areas. The large central domains of MaSp1 and MaSp2 are organized in block copolymer-like arrangements, in which two basic sequences, crystalline [poly(A) or poly(GA)] and less crystalline (GGX or GPGXX) polypeptides alternate in core domain. The main difference between MaSp1 and MaSp2 is the presence of proline (P) residues accounting for 15% of the total amino acid content in MaSp2 (Hu *et al*., 2006), whereas MaSp1 is proline-free. By calculating the number of proline residues in *N. clavipes* dragline silk, it is possible to estimate the presence of the two proteins in fibres; 81% MaSp1 and 19% MaSp2 (Brooks *et al*., 2005). Different spiders have different ratios of MaSp1 and MaSp2. For example, a dragline silk fibre from the orb weaver *Argiope aurantia* contains 41% MaSp1 and 59% MaSp2 (Huemmerich *et al*., 2004). Such changes in the ratios of major ampullate silks can dictate the performance of the silk fibre (Vollrath and Knight, 1999). Specific secondary structures have been assigned to poly(A)/(GA), GGX and GPGXX motifs including β-sheet, 3_10_-helix and β-spiral respectively (Humenik *et al*., 2011). The primary sequence, composition and secondary structural elements of the repetitive core domain are responsible for mechanical properties of spider silks; whereas, non-repetitive N- and C-terminal domains are essential for the storage of liquid silk dope in a lumen and fibre formation in a spinning duct (Ittah *et al*., 2006). The primary amino acid sequence, composition and secondary structural elements of other silk types are reviewed elsewhere (Lewis, 2006b; Humenik *et al*., 2011).

### Production of recombinant silk proteins

Spiders cannot be farmed, in contrast to silkworms, due to their aggressive behaviour and territorial nature (Kluge *et al*., 2008). Collecting silk from webs is a time-consuming task. It took 8 years to make a golden spider silk cape from 1.2 million golden orb webs (Chung *et al*., 2012). Therefore, biotechnological production of recombinant spider silks is the only practicable solution to harvest silks on a larger scale and to meet growing needs of medicine and biotechnology. A variety of heterologous host systems have been explored to produce different types of recombinant silks (Table 1 and Table 2). Recombinant partial spidroins as well as engineered silks have been cloned and expressed in bacteria (*Escherichia coli*), yeast (*Pichia pastoris*), insects (silkworm larvae), plants (tobacco, soybean, potato, Arabidopsis), mammalian cell lines (BHT/hamster) and transgenic animals (mice, goats).

**Table 1:** Summary of recombinantly expressed spider silks in bacteria and yeasts. Spider silk origin, number of monomers, molecular weight, cloning and expression plasmids as well as restriction enzymes and purification strategies used to produce recombinant silks are shown

Type	Host	Origin	Protein	Number of monomers	MW (KDa)	Cloning plasmid	RE	Expression plasmid	Purification Strategy	Yield (mg L^−1^)	References
Bacteria	*E. coli*	*Nephila clavipes*		16, 32, 64, 96	55, 100, 193, 285	pET30a(+)	*Nhe I/ Spe I*	pET30a(+)	Ammonium sulphate		Xia *et al*., 2010
				6; 15	16, 39.5			pETNX	PA	96.8 mg L^−1^; 200 mg L^−1^	Dams-Kozlowska *et al*., 2012
			MaSp 1	8; 16	65–163	pBR322 derived	*Pst I*	pFP202 (pET9a + pET11a)		300 mg L^−1^	Fahnestock and Irwin, 1997
				8; 16	65–163			pFP202, pFP204, or pFP207	IMAC		Fahnestock and Irwin, 1997
				16, 24	46, 70	pBSSKII+	*AvrII, Nhe1*	pET19k		N A^−1^	An *et al*., 2011
			poly(A) and GGX	1, 2, 3, 6	10, 18	pET30a(+)	*Spe I/Nde I*	pET30a(+)		25 mg ml^−1^	Rabotyagova *et al*., 2009
	*E. coli*	*Nephila clavipes*		8; 16	65–163	pBR322 derived	*Pst I*	pFP202, pFP204, or pFP207		300 mg L^−1^	Fahnestock and Irwin, 1997
				8, 16, 32	31, 58, 112	pBBSK	*Sca/Xma/BspEI*	pET19b		10 mg g^−1^	Lewis *et al*., 1996
		*Argiope aurantia*	MaSp2	16	63				IMAC		Brooks *et al*., 2008
				12	71	pET30a(+)	N A^−1^	pET30a(+)		N A^−1^	Brooks *et al*., 2008
				8	67						Brooks *et al*., 2008
	*E. coli*	*Nephila clavipes*	Masp1/Masp2	24/16	62/47	pBSSKII+	*Xma1/Sca1/BspE1*	pET19K	IMAC	120 mg L^−1^	An *et al*., 2011
				1x-18x	15, 23, 32, 41	pUC18	*Spe I/Nde I*	pQE-9		15, 7, 3, 2 mg L^−1^	Prince *et al*., 1995
	*E. coli*	*Argiope trifasciata*	AcSp1	2, 3, 4	19, 38, 51.7, 76.1	pET32	*BamHI/ BsgI/ BseRI*	pET32	IMAC	80 mg L^−1^; 22 mg L^−1^	
		*Nephila antipodiana*	TuSp1	11	190		Xma1/Pvu1/BspE1			40 mg L^−1^	Xu *et al*., 2007
	*Salmonella*	*Araneus diadematus*	ADF1	1x-3x	30–56	pJ2	*HindIII/XbaI*	pTRC99a_Cm	SEC	N A^−1^	Widmaier *et al*., 2009
			ADF2	1x-3x							Widmaier *et al*., 2009
			ADF3	1x-3x							Widmaier *et al*., 2009
Yeast	*Pichia Pastori*	*Nephila clavipes*	Masp 1	8, 16	65	pBR322 derived	*Pst I*	pFP684	Ammonium sulphate	663 mg L^−1^	Fahnestock and Bedzyk, 1997

**Table 2:** Summary of recombinantly expressed spider silks in insects, plants and mammalians. Spider silk origin, number of monomers, molecular weight, cloning and expression plasmids as well as restriction enzymes and purification strategies used to produce recombinant silks are shown

Type	Host	Origin	Protein	Number of monomers	MW (KDa)	Cloning plasmid	RE	Expression plasmid	Purification strategy	Yield (mg L^−1^)	References
Insects	*B. mori*	*Nephila clavipes*	Masp1	2	83	pSLfa1180fa	*Spe1/Nde1*	pBac[3xP3-DsRedaf]	IMAC	N A^−1^	Wen *et al*., 2010
				4	70	pSL1180		pFastBacHT-C		6 mg/larva	Zhang *et al*., 2008
			Masp1+Flag	multiple	75–130	pBSSKII+ and pSLfa1180fa	*Sca1/Xma1/BspE1*	pBAC[3xP3-DsRedaf]		N A^−1^	Teulé *et al*., 2012b
Plants	*Nicotiana tobaccum Solaum tubercum*		Masp1	multiple	12.9–99.8	pUC19	*NgoMIV/HindIII*	pRTRA7/3	(NH_4_)_2_SO_4_ 10–50% saturation	0.5-2 % total protein	Scheller *et al*., 2001
	*Nicotiana benthamiana*	*Nephila clavipes*	Flag (intein)	4; 10	47, 72, 100, 250	pRTRA15	splicing events	pCB301-Kan	IMAC	1.8 mg/50 g leaft material 0.34%; 0.03% in leaves, 1.2%; 0.78% in seeds	Hauptmann *et al*., 2013
	*Arabdopsis thaliana Glycine max*		Masp1	8, 16	64, 127	pBSSK+	*BglII/BamH1*	Cong' + Pha3'	ammonium sulphate	1% in somatic embryos	Barr *et al*., 2004
Mammalians	Trangenic mice	*Nephila clavipes*	MaSP1	6	31–66	pGEM-5zf	*BamHI/NcoI*	pBC1	centrifugation	11.7 mg L^−1^	Xu *et al*., 2007
	COS-1 cells	*Euprosthenops sp.*			25, 22	pER1-14	*BamH1/EcoRV*	pSecTag2/Hygro A	N A^−1^	N A^−1^	Grip *et al*., 2006
	Baby hamster kidney	*Nephila clavipes*	Masp1/ Masp2	N A^−1^	59, 106/ 59	pBSSK+	*ApaI/SapI*	CMV promoter	ammonium sulfate		
	Baby hamster kidney	*Araneus diadematus*	ADF3		63, 60, 110, 140	pSecTag-C	*MscI/PvuII*			25–50 mg L^−1^	Lazaris *et al*., 2002

#### Unicellular organisms as heterologous host systems

**Figure 2:**
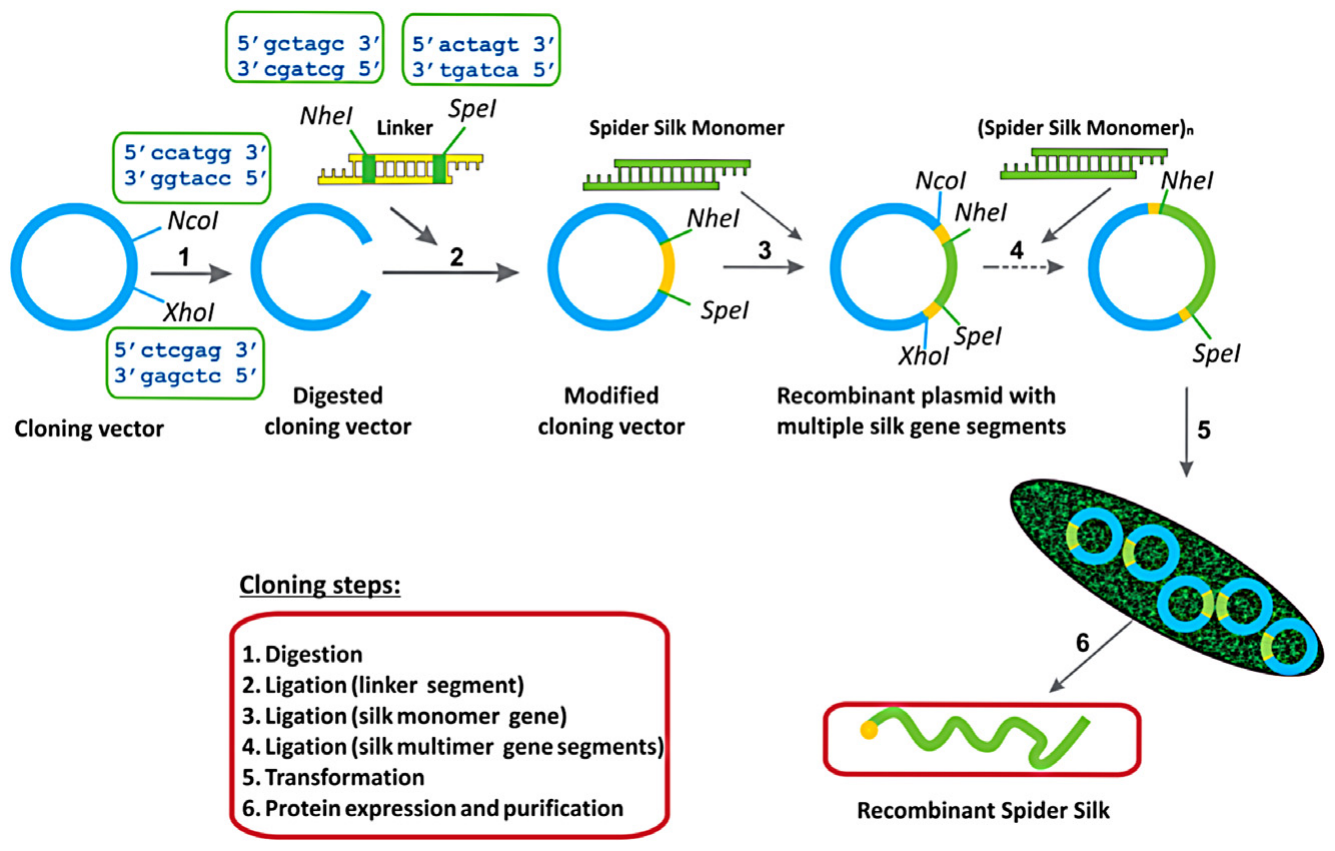
Recombinant DNA approach used to prepare silk-like proteins.

Unicellular organisms, such as bacteria and yeast, have been investigated as host systems for recombinant silks. A gram-negative, rod-shaped bacterium *E. coli* is a well-established host for industrial scale production of proteins. Therefore, the majority of recombinant spider silks have been produced in *E. coli* (Lewis *et al*., 2011; Fahnestock and Irwin, 1997; Wang *et al*., 2006; Rabotyagova *et al*., 2009; Rabotyagova *et al*., 2010; An *et al*., 2011; An *et al*., 2012; Teulé *et al*., 2012a). *E. coli* is easy to manipulate, has a short generation time, is relatively low cost and can be scaled up for larger amounts protein production. The recombinant DNA approach enables the production of recombinant spider silks with programmed sequences, secondary structures, architectures and precise molecular weight (Rabotyagova *et al*., 2011). There are four main steps in the process: (i) design and assembly of synthetic silk-like genes into genetic ‘cassettes’, (ii) insertion of this segment into a DNA vector, (iii) transformation of this recombinant DNA molecule into a host cell and (iv) expression and purification of the selected clones. Figure 2 summarizes the recombinant DNA approach used to prepare silk-like proteins.

**Figure 3:**
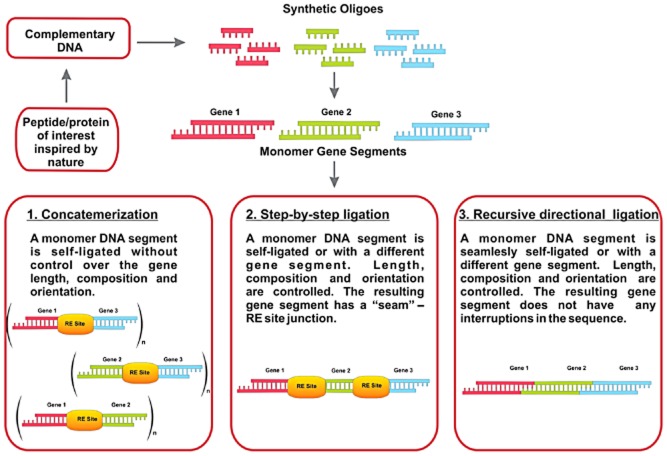
Gene multimerization approaches. Note: RE Site stands for a restriction enzyme site.

The monomeric silk-like gene sequences can be synthesized as short single-stranded oligonucleotides (up to 100 bp) by commercial oligonucleotide synthesis or used directly as polymerase chain reaction products from cDNA libraries. Large repetitive sequences can be constructed by using concatemerization, step-by-step directional approach and recursive ligation (Fig. 3). Concatemerization is a useful method when a library of genes of different sizes is desired but has limitations in the preparation of genes with specific sizes (Meyer and Chilkoti, 2002). To overcome limitations of concatemerization, recursive directional ligation or a step-by-step ligation is employed (Meyer and Chilkoti, 2002; Wright and Conticello, 2002). Recursive directional ligation allows for facile modularity, where control over the size of the genetic cassettes is achieved. Moreover, recursive directional ligation eliminates the restriction sites at the junctions between monomeric genetic cassettes without interrupting key gene sequences with additional base pairs that makes it different from the step-by-step ligation approach (Higashiya *et al*., 2007).

For example, we have employed step-by-step directional ligation to produce various partial recombinant spider silks as well as engineered silk-like proteins based on the sequences of dragline silk originated from *N. clavipes* (Prince *et al*., 1995; Wang *et al*., 2006; Huang *et al*., 2007; Rabotyagova *et al*., 2009; 2010; Mieszawska *et al*., 2010; Gomes *et al*., 2011; Numata *et al*., 2012). As one example, spider silk block copolymers were generated in *E. coli* (Rabotyagova *et al*., 2009; 2010). In the first cloning step, a commercially available pET30a(+) vector (Novagen, San Diego, CA, USA) was modified with an adaptor sequence, carrying *NheI* and *SpeI* restriction sites. The adaptor was inserted into *XhoI* and *NcoI* sites of a pET30a(+) to generate pET30L. The coding sequences of two spider silk-like monomers A (hydrophobic block) and B (hydrophilic) were designed to carry *SpeI* and *NheI* restriction sites at the ends of the sequences. This allowed ligation of the domains into a pET30L vector. By using a step-by-step directional ligation approach, direct control over the assembly of monomeric genes into complex sequences was achieved. Six different constructs were cloned and transformed into the bacterial host for expression. An N-terminal His-tag was used for protein purification by immobilized metal affinity chromatography (Rabotyagova *et al*., 2009).

**Figure 4:**
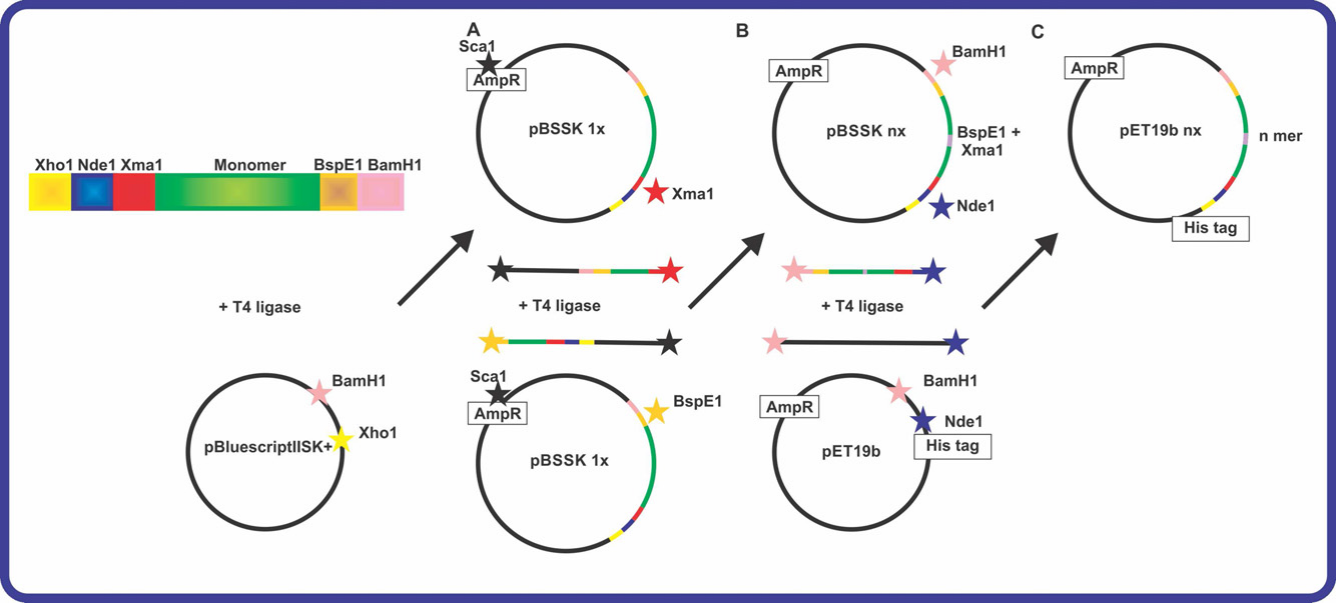
Cloning strategy used by the Lewis group to engineer long repetitive spider silk sequences (in green). A. Cloning of a silk monomer into the vector pBluescript II SK+. B. The resulting plasmid is double digested and fragments containing silk monomers are ligated again to produce longer sequences. C. The synthetic spider silk multimer is ligated into pET19b expression vector. Note: Restriction digestion sites are indicated by star. Adapted from reference (Teule *et al*., 2009).

Another genetic engineered strategy has been proposed by Lewis Laboratory to assemble long repetitive spider silk genes (Teule *et al*., 2009). This cloning strategy employs a one-step head-to-tail ligation that can produce large inserts in precise manner (Lewis *et al*., 1996; Brooks *et al*., 2008; Teule *et al*., 2009; Teulé *et al*., 2012a). The spider silk synthetic genes were optimized for codon usage in *E. coli* and were cloned into a plasmid vector pBluescriptII SK(+) (Stratagene). Each silk module was carrying compatible *XmaI* and *BspEI* restriction sites at the ends on the coding sequences. The vector also contained a unique restriction site (*ScaI*) in the ampicillin resistance gene. By simultaneously performing two double digestion reactions *ScaI – XmaI* and *ScaI* – *BspEI* two fragments each containing a copy of a silk monomer gene were obtained. The fragments were ligated together using T4 ligase resulting in the doubling of the size of silk genes and restoring the ampicillin resistance of the plasmid (Fig. 4). Several round of cloning were performed to obtain repetitive sequences of a desired size. Next, the multimeric synthetic genes were subcloned into an expression pET19b vector using *NdeI* and *BamHI* restriction sites. Since the expression vector was carrying *NdeI* and *BamHI* sites, the liberated inserts were cloned in-frame with pET19b. Similar to pET30L, silk genes in pET19b are under control of the T7 promoter and require the addition of isopropyl-β-D-1-thiogalactopyranoside to initiate protein expression. The expressed proteins can be purified by immobilized metal affinity chromatography (IMAC) due to the presence of an N-terminal His-tag. Several recombinant spider silk proteins from different species were produced using this genetic engineering strategy including silks from *N. clavipes* (Teule *et al*., 2009) *Argiope aurantia* (Brooks *et al*., 2008). Recombinant spider silk proteins from *Nephylengys cruentata*, *Parawixia bistriata* and *Avicularia juruensis* were produced employing this cloning strategy (Leopoldo *et al*., 2007) (US patent 20 100 311 645). Figure 4 summarizes the strategy.

A three module cloning strategy based on the sequences of ADF-3 and ADF-4 was developed by Scheibel research group (Huemmerich *et al*., 2004), designed so that multiple modules can be combined. Moreover, additional coding sequences such as N- or C-terminal domains can be added if needed. The purification protocol is based on heat resistance of silk proteins followed by an ammonium sulphate precipitation that is different from Ni-NTA IMAC.

Different purification strategies have been employed recently to optimize small and large-scale production of recombinant silks. Most of the spider silk proteins are produced with an N- or C-terminal His-tags to make purification simple and produce enough amounts of the protein. However, the presence of this tag can affect protein secondary structure and interfere with the process of spider silk fibre formation. Dams-Kozlowska *et al*. (2012) proposed two strategies to purify spider silks from lysates without the use of a His-tag. These protocols are based on thermal treatment and organic acid resistance of silk proteins and do not require the presence of the His-tag. After purification, silk proteins based on MaSp1 gene sequence were formed into films that subsequently were used to grow murine fibroblast cell culture. The results demonstrated that silk films were non-toxic to the cells (Dams-Kozlowska *et al*., 2012).

Because of the highly repetitive core sequence of spider silk genes, frequent homologous recombination, deletions, transcription errors, translation pauses, accumulation in inclusion bodies and low yields were observed during the production of recombinant silks in *E. coli*. Moreover, when the protein size was increased from 43 kDa to higher (the size of native spidroins is between 300 and 350 kDa), protein yields decreased dramatically. Codon optimization for the specific host expression system helped maximize the translation of the foreign gene transcripts and thus, improved protein yields (Fahnestock and Bedzyk, 1997, Lewis, 2006b). It was also suggested that depletion of tRNA pools upon protein expression resulted in transcription and translation errors (Rosenberg *et al*., 1993). Recently, Xia *et al*. (2010) employed a metabolic engineered strategy to enhance the production of recombinant spider silks. The authors reported production of full length (284.9 kDa) recombinant *N. clavipes* dragline silk proteins that were rich in glycine (43–45%). Production of these silk proteins was enhanced by the use of the metabolically engineered expression host within which the glycyl-tRNA pool was elevated. The fibres spun with the native-sized recombinant spider silk protein showed tenacity, elongation and Young's modulus of 508 MPa, 15% and 21 GPa, respectively, comparable to those of native spider dragline silk (Xia *et al*., 2010). Through extensive proteomic analysis, serine hydroxymethyltransferase (GlyA) and β-subunit of glycly-tRNA synthetase (GlyS) were found to be upregulated to meet the high cellular demand for glycly-tRNA when expressing glycine-rich silk proteins. Increased glycine biosynthetic flux by overexpressing glycyl-tRNA synthetase elevated the total tRNAGly pool and resulted in enhanced production of high molecular weight recombinant spider silks.

Recently, large spider recombinant egg case silk protein from *Nephila antipodiana*, 378 kDa, was engineered using *E. coli*, where gene multimers were chemically linked by cysteine disulfide bonds. The recombinant silk sequence consisted of two silk proteins: tubuliform spidroin 1 (TuSp1) and C-terminal domain of MisP1. Non-repetitive C-terminal domain of MiSp1 was chosen due to its higher water solubility and stability compared with the C-terminal domain of TuSp1. A disulfide linkage between two C-terminal domains was formed by introducing a point mutation (S76 to S76C). This link allowed the formation of a hybrid DNA construct that was expressed in *E. coli* (DE3). The recombinant protein was expressed in *E. coli*. Moreover, the artificial fibres spun from this protein showed higher tensile strength and Young' modulus than natural egg case protein (Lin *et al*., 2013).

The highly repetitive silk gene arrangement and the unusual mRNA secondary structure result in inefficient translation that limits the size of the silks produced in *E. coli*. To minimize the presence of truncated silk proteins and allow the extracellular secretion of silks, the mythylotropic yeast *P. pastoris* has been used. Fahnestock and Bedzyk (1997) produced *N. clavipes* spider dragline silks in yeast *P. pastoris*. Synthetic genes were expressed at high levels under control of the methanol-inducible *AOX1* promoter. Transformants containing multiple gene copies produced elevated levels of silk protein. Results demonstrated that *P. pastoris* can be used to successfully produced produce long repetitive proteins (Fahnestock and Bedzyk, 1997).

Spider silks from *Araneus diadematus* (ADF-1, 2 and 3) have also been expressed using the type III secretion system of a gram-negative, non-spore-forming, enterobacterium *Salmonella*. The authors reported yield values range from 90 to 410 nmol L^−1^h^−1^ that is similar to 10 mg L^−1^ h^−1^ for a protein the size of ADF-2. The results demonstrated the feasibility to use *Salmonella* for the large-scale spider silk production (Widmaier *et al*., 2009). Mammalian cell lines, such as bovine mammary epithelial alveolar and baby hamster kidney cells, were used to express MaSp1 and MaSp2 (Lazaris *et al*., 2002). The cells expressed recombinant proteins; however, as size of silk gene increased, the yield decreased dramatically due to inability of mammalian cells to cope with large repetitive sequences. Several factors have attributed to the decreased yields including, but not limited to, inefficient transcription, insufficient secretion, low copy numbers and translational limitations. The produced silk proteins were spun into fibres, and their mechanical properties were tested. It was noted that those recombinant silks that were produced without a His-tag demonstrated better mechanical properties compared with fibres made of silk proteins with a His-tag (i.e. fibres were brittle). Similar problems (i.e. transcription and translation limitations) have been reported when green monkey kidney fibroblast-like cell lines (COS-1) were used to express a 636-base pair gene fragment of MaSp1 from the African spider *Euprosthenops sp*. (Grip *et al*., 2006). Table 1 summarizes genetic engineering approaches, cloning strategies, and production yields of recombinant silk proteins produced in unicellular heterologous host systems.

#### Multicellular organisms as heterologous host systems

Due to the low production rate and instability (i.e. frequent homologous recombination, deletions, transcription errors, translation pauses) of spider silk repetitive genes in unicellular organisms, multicellular organisms such as insects, plants and mammals have been studied for production of recombinant spider silk proteins.

Silkworms (*B. mori*) can be farmed and produce cocoons containing large quantities of silkworm silk known as fibroin (Vepari and Kaplan, 2007; Hu and Kaplan, 2011). Moreover, to produce a solid thread, silkworms employ a spinning process that is similar to that used by spiders to make dragline silk. The presence of a natural silk production system in silkworms makes them excellent candidates to investigate as heterologous hosts for spider silk production. There have been several reports of the transfer of silk genes from spiders to silkworms (Motohashi *et al*., 2005; Zhang *et al*., 2011; Teulé *et al*., 2012b).

Baculovirus-based expression systems have been used to introduce silk genes into a heterologous host. Baculovirus infects silkworms and allows for production of large quantities of heterologous proteins in a short period of time (Motohashi *et al*., 2005). Using this expression system, MaSp1 from *N. clavipies* linked with an enhanced green fluorescent protein (EGFP) fusion protein was cloned and expressed in the *B. mori* cell line (BmN) and larvae (Zhang *et al*., 2008). The authors reported successful production of a recombinant EGFP-MaSp1 fusion protein in both systems. In the silkworm larvae, a total of 6 mg of fusion protein was expressed, whereas in the BmN cells, 5% of the cell total protein was occupied by this recombinant silk. The major limitations of this expression system were low solubility of silk proteins and inability to assemble spider silk fibres. It was shown that more than 60% of the fusion proteins formed aggregates via self-assembly. To overcome solubility issues, MaSp1 C-terminal domain is to be incorporated due to its role to prevent aggregate formation. To produce fibres, germline-transgenic silkworms (*B. mori*) were produced by injecting silkworm eggs with a *piggyBac* transformation vector carrying MaSp1 sequence (Wen *et al*., 2010). The insects were capable of spinning fibres and forming cocoons containing recombinant spider silk. However, the mechanical properties of the fibres were lower than dragline MaSp1 silk due to the low ratio of MaSp1 in the total silk protein.

**Figure 5:**
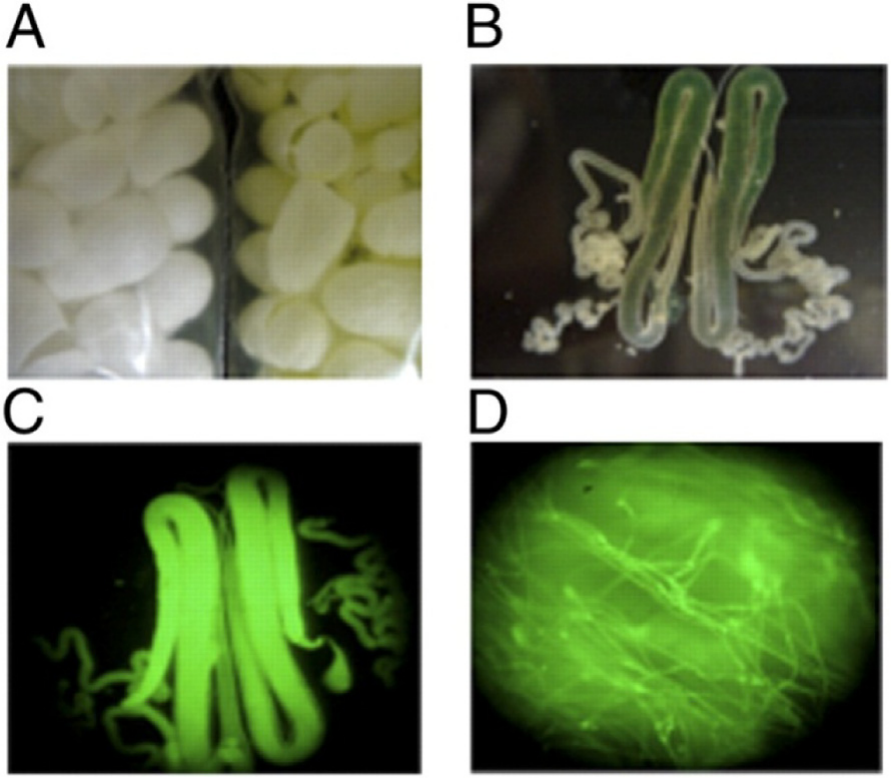
Expression of the chimeric silkworm/spider silk/EGFP protein in (A) cocoons, (B and C) silk glands and (D) silk fibres from spider 6-GFP silkworms. Reproduced with permission from (Teulé *et al*., 2012b).

In a recent effort to develop tough fibres, transgenic silkworms encoding chimeric silkworm/spider silk proteins were produced using *piggyBac* vectors (Teulé *et al*., 2012b). The vector, used previously by the Tamada group (Kojima *et al*., 2007) included the *B. mori* fibroin heavy chain promoter and enhancer, a genetic sequencing encoding a 78 kDa synthetic spider silk protein, and an EGFP tag. Strong EGFP signals were observed by fluorescence (Fig. 5). The composite fibres were tougher than the parental silkworm silk fibres and as tough as native dragline spider silk fibres.

These results demonstrate that silkworms can be engineered to generate composite silk fibres containing stably integrated spider silk protein sequences, which significantly improved overall mechanical properties.

Transgenic plants have also been investigated as heterologous host systems to produce recombinant spider silks. Advances in genetic engineering technology and transformation methods make it possible to produce non-plant proteins in plants (Yang *et al*., 2005; Rech *et al*., 2008). Moreover, one plant offers several different expression systems, such as seeds, leaves, tubers and roots with potential for organelle-specific accumulation of recombinant proteins (Scheller and Conrad, 2005).

Stable transgenic tobacco and potato lines were engineered to express *MaSp1* genes from *N. clavipes* ranging from 420 to 3600 bp (Scheller *et al*., 2001). Recombinant spider silk proteins were found in the endoplasmic reticulum (ER) of tobacco and potato leaves at the accumulation of 2% of total soluble protein. Moreover, the production levels were independent of the size of silk genes. Purification was performed using high temperature treatment followed by acidification and ammonium sulphate precipitation. Additionally, recombinant MaSp1-like proteins were also produced in the leaves and seeds of *Arabidopsis* (small flowering plants related to cabbage) as well as in somatic soybean embryos (Barr *et al*., 2004). The expression of recombinant silks was driven by the 35S promoter in leaves and the β-conglycinin α' subunit promoter in seeds and somatic soybean embryos. The results demonstrated that recombinant spider silk proteins had higher accumulation levels in seeds than in the leaves. Recently, a native-sized FLAG protein from *N. clavipes* was cloned and expressed in the ER of tobacco plant (*Nicotiana benthamiana*) leaf cells using an intein-based posttranslational protein fusion technology (Hauptmann *et al*., 2013). This method avoids the need for highly repetitive transgenes resulting in a higher genetic and transcriptional stability. Additional details on production of fibrous proteins in plants can be found elsewhere (Scheller and Conrad, 2005).

Transgenic production of recombinant silk proteins in mammary glands and secretion of them into milk has been investigated in mice and goats (Williams, 2003; Xu *et al*., 2007). In case of transgenic mice production, MaSp1 and MaSp2 synthetic genes (40 and 55 kDa) were synthesized and cloned into the pBC1 expression vector (Invitrogen, Carlsbad, CA, USA) together with a goat β-casein signal sequence. The chimeric gene construct was microinjected into pronuclei of fertilized eggs of Kunming white mice (Xu *et al*., 2007). Southern blot analysis was used to identify mice containing transgene construct as well as a copy number of transgene. The expression of dragline silk in milk was confirmed by Northern blot followed by Western blot analysis. The results revealed that transgenic mice were capable of expressing recombinant silk proteins in their milk. Genetically engineered (transgenic) goats capable of expressing spider silk proteins based on the sequences of MaSp1 and MaSp 2 were produced by Nexia Biotechnologies, and later by the Lewis group (Lazaris *et al*., 2002; Service, 2002). Silk protein expression was controlled by the β-casein promoter and was expressed in the milk of transgenic goats. Silk proteins were observed only in mammary tissues as confirmed by Western blot (Steinkraus *et al*., 2012). Maximum yields observed for the recombinant silk production in transgenic animals were low (11.7 mg l^−1^) when compared with bacterial expression (Table 1 and Table 2). Today, the large-scale production of recombinant silk proteins from transgenic animals is relatively expensive and challenging in terms of animal breeding.

## Future outlook

Over the last decade there has been considerable progress in understanding the genetic organization encoding spider silks. Cloning, expression and purification of spider silks has improved, and the self-assembly and processing of spider silk into many material formats is now better understood. Recently a native-sized (285 kDa) recombinant protein of the spider *N. clavipes* was produced and spun into a fibre displaying mechanical properties comparable to those of the native silk, indicating a breakthrough in standard recombinant production of spider silks. Moreover, a variety of heterologous host systems have been explored to produce different types of recombinant silks. For example, transgenic silkworm/spider silk production systems have been developed to produce tough fibres. It is possible to mix and match key modules via recombinant approaches, providing additional insights into the role of individual modules and effects of neighbouring elements on properties. This approach should lead to the development of custom structures built from specific silk elements. Future challenges will include development of tailor-made production systems for recombinant silks keeping in mind differences in chemical and physical properties of individual silk modules, scaling up silk production, prevention of the formation of aggregates and matches to the mechanical properties of silk fibres.
